# PM2.5-induced cardiovascular dysregulation in rats is associated with elemental carbon and temperature-resolved carbon subfractions

**DOI:** 10.1186/1743-8977-11-25

**Published:** 2014-05-22

**Authors:** James G Wagner, Ali S Kamal, Masako Morishita, J Timothy Dvonch, Jack R Harkema, Annette C Rohr

**Affiliations:** 1 Department of Pathobiology and Diagnostic Investigation, Michigan State University, East Lansing, MI, USA; 2 Center for Integrative Toxicology, Michigan State University, East Lansing, MI, USA; 3 Human Exposure and Atmospheric Sciences Division, NERL, U.S. Environmental Protection Agency, Research Triangle Park, NC 27711, USA; 4 Department of Environmental Health Sciences, School of Public Health, University of Michigan, Ann Arbor, MI, USA; 5 Electric Power Research Institute, Palo Alto, CA, USA; 6 Michigan State University, 1129 Farm Lane, Rm211, East Lansing, MI 48824, USA

**Keywords:** Air pollution, Blood pressure, Elemental carbon, Heart rate, Inhalation, Organic carbon, PM_2.5_, Rat

## Abstract

**Background:**

We tested the hypothesis that cardiovascular responses to PM_2.5_ exposure will be enhanced in hypertensive rats and linked to specific carbonaceous pollutants in an urban industrial setting.

**Methods:**

Spontaneously hypertensive rats were exposed by inhalation to concentrated PM_2.5_ in an industrial area of Dearborn, Michigan, for four consecutive summer days. Blood pressure (BP), heart rate (HR) and HR variability (HRV) metrics (SDNN, RMSSD) were assessed by radiotelemetry and compared to 1 h- and 8 h-averaged fluctuations in PM_2.5_ composition, with a focus on elemental and organic carbon (EC and OC, respectively), and temperature-resolved subfractions (EC1-EC5, PC (pyrolized carbon), and OC1-OC4), as well as other major and minor PM components.

**Results:**

Mean HR and BP were increased, while HRV was decreased over 4 days of exposure. Using 1 h averages, EC (1 μg/m^3^ increase) was associated with increased HR of 11-32 bpm (4-11% increase), 1.2-1.5 ms (22-27%) decreases in SDNN, 3-14 mmHg (1.5-8%) increases in systolic BP, and 5-12 mmHg (4-9%) increases in diastolic BP. By comparison, associations with OC were negligible. Using 8 h averages, EC subfractions were linked with increased heart rate (EC1: 13 bpm; EC2, EC3, PC: <5 bpm) and SDNN (EC1> > EC2 > EC3, EC4, PC), but with decreased RMSSD (EC2, EC5 > EC3, EC4). Minimal effects were associated with OC and OC1. Associations between carbon subfractions and BP were negligible. Associations with non-carbonaceous components and trace elements were generally non-significant or of negligible effect size.

**Conclusions:**

These findings are the first to describe associations between acute cardiovascular responses and thermally resolved carbon subfractions. We report that cardiovascular responses to PM_2.5_ carbonaceous materials appear to be driven by EC and its EC1 fraction.

## Background

Associations of ambient fine particulate matter (PM_2.5_) with acute myocardial events, including ischemia, stroke, arrhythmia, and heart failure exacerbation, are well documented [1]. An increased risk for ischemic cardiac events of 4-20% have been associated with an increase of 10 μg/m^3^ in ambient PM_2.5_[2,3]. Biological mechanisms for these associations are unknown, but altered vascular reactivity and cardiac function that have been observed during PM_2.5_ exposure may be critical, initial responses that lead to more serious myocardial events. Community-based studies in diverse airsheds such as Boston, MA, Detroit, MI and Beijing, China demonstrate significant associations between daily variations in PM_2.5_ and increases in systolic blood pressure (BP) [4-6]. In addition, exposure-related changes in heart rate variability (HRV) have been observed in diabetics and cardiac patients in relation to ambient PM_2.5_[7,8], and in healthy elderly volunteers with concentrated PM_2.5_[9]. While such acute responses may be harmless in otherwise healthy subjects, people with preexisting cardiovascular, respiratory or metabolic conditions may suffer more significant and deleterious consequences [10].

In addition to associations with PM_2.5_ mass, two recent meta-analyses using Medicare records and particle composition data from the Environmental Protection Agency Chemical Speciation Network found significant associations between elemental carbon (EC), a combustion product most often linked to diesel engine exhaust, and hospital admissions for cardiovascular causes [11,12]. Ambient EC concentrations have been associated with increased systolic pressure [13-15] and HRV [13,16]. Another major carbonaceous fraction of PM_2.5_ is organic carbon (OC), which is derived from mobile sources, biomass burning and industrial processes and has also been linked to decreased HRV and elevated BP [6,16]. Extensive analyses of PM_2.5_ EC and OC using thermal/optical analytical approaches yields up to five OC subfractions (OC1-OC4 and pyrolized carbon [OP]) and four EC subfractions (EC1-EC4). This method applies step-wise increases in temperature in a controlled atmosphere to oxidize first organic and then elemental carbon samples to yield eight fractions with decreasing volatility and increasing structural complexity. Thus OC1 and EC1 are more volatile and simpler in structure than OC4 and EC4, respectively. These carbon subfractions have been used to refine source apportionment analysis in the U.S. [17] and more recently in China [18,19]; however, the relative health effects of these temperature-resolved subfractions of EC and OC have not been evaluated.

We have previously reported altered HRV in hypertensive rats exposed to concentrated PM_2.5_ in Detroit, MI and Steubenville, OH that was linked to specific PM components and related industrial and mobile sources [20,21]. In these studies we found EC to consistently have the strongest association of any PM_2.5_ component with changes in heart rate and HRV. Our goal in the present study was to extend these observations to include blood pressure responses in hypertensive rats, as well as provide a detailed characterization of both EC and OC and their subfractions in an industrial urban center in Dearborn, MI. Our results describe clear differences between EC and OC in PM2.5-induced responses, as well as provide initial insights into the relative potencies of carbon subfractions in relation to acute cardiovascular responses.

## Results

### Exposure characterization

**Figure 1:**
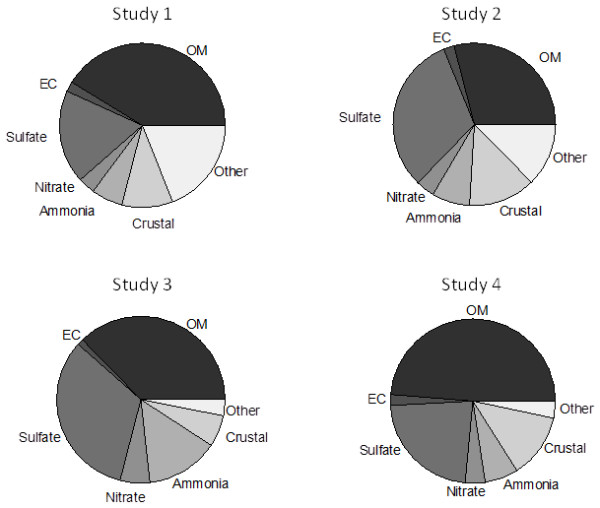
**Distribution of major components in concentrated PM**_**2.5 **_**during exposures.** Concentrations of organic matter (OM: OC × 1.8), elemental carbon (EC), sulfate, nitrate, ammonia, crustal elements and other components were determined from 8 h samples from the four separate field studies as described in Materials and Methods. Crustal = 2.14Al + 2.43Fe + 1.54Si, where Si was estimated as potassium (K)/0.15.

**Figure 2:**
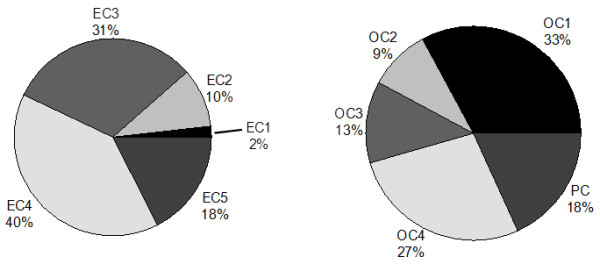
**Distribution of EC and OC subfractions during exposures.** Concentrations in 8 h samples of subfractions of elemental carbon (EC) and organic carbon (OC) were determined by thermal/optical approaches as described in Materials and Methods.

The average (±SD) chamber PM_2.5_ mass concentrations during the four 4-day exposure studies were as follows: Study 1, 415 ± 99; Study 2, 642 ± 294; Study 3, 767 ± 256; Study 4, 364 ± 58 μg/m^3^. Figure 1 depicts the distribution of major chemical components of PM_2.5_ collected during each 4-day inhalation exposure period. Table 1 shows the combined numeric average for all exposures. As previously observed in this Dearborn study area [22,23], sulfate and OC dominated during the summer months. Figure 2 shows the average carbon fraction distribution of concentrated PM_2.5_ during the exposures. EC3 & EC4 were the most prevalent EC subfractions, while OC1 & OC4 subfractions were the most prevalent species within OC.

**Table 1:** **Average mass of PM**_**2.5 **_**constituents in chambers during inhalation exposures**

**Constituent**	**Mass (SEM) (mg/m3)**
PM2.5	547 ± 62.3
OM	208 ± 15.3
EC	10 ± 1.3
Sulfate	154 ± 28.8
Nitrate	24 ± 5.5
Ammonium	52 ± 11.9
Urban dust	56 ± 8.1
Other	58 ± 14.7

Data represent the daily mean of 16 days of exposures (four studies of 4 days each). OM-organic matter; EC – elemental carbon.

### Effect of PM_2.5_ exposure on cardiovascular responses

Comparisons of responses in rats exposed to AIR vs. PM_2.5_ using 1 h averaged data resulted in several-exposure-related differences (Table 2). Changes in blood pressure responses were the most sensitive indicator of PM_2.5_ exposure, with significant associations with MAP, systolic and diastolic pressures during 3 of the 4 exposure studies. Other significant associations were found for heart rate during studies 1 & 3, and SDNN during Study 3.

**Table 2:** **Effect of PM**_**2.5 **_**exposure on cardiovascular responses in SH rats**

**Response**	**Study**	**dfN**	**dfD**	**p-value**
**Heart rate**	1	35	421	*0.0246
	2	35	455	0.365
	3	35	493	*0.0008
	4	35	504	0.661
**lnSDNN**	1	35	421	0.591
	2	35	455	0.061
	3	35	493	*0.0372
	4	35	504	0.356
**lnRMSSD**	1	35	421	0.076
	2	35	455	0.546
	3	35	493	0.289
	4	35	504	0.266
**MAP**	1	35	391	*0.0124
	2	35	456	*0.0107
	3	35	495	*0.0106
	4	35	504	0.459
**Systolic**	1	35	391	*0.0239
	2	35	456	*0.0127
	3	35	495	*0.0193
	4	35	504	0.476
**Diastolic**	1	35	391	*0.0085
	2	35	456	*0.0097
	3	35	495	*0.0059
	4	35	504	0.415

Data are expressed as p-values for the interaction of Exposure (Air vs. PM_2.5_) and time (1 h intervals). dfN-degrees freedom for subjects, dfD degrees freedom for observations. *p < 0.05.

### Associations with major components and trace elements

Assessment of major non-carbonaceous PM components (nitrates, sulfates and ammonia) and 29 elements revealed a number of minor, but statistically significant associations with HR, HRV and BP (Table 3, Additional file 1: Tables S1 and S2). For example, significant responses of less than 0.1 bpm for HR and less than 0.01 ms for lnSDNN were associated with the change in IQR for a few specific components or elements. The only PM_2.5_ constituent with a larger effect estimate was uranium, which was associated with a 15 mm Hg increase in diastolic pressure (Table 3), although the associations between this element and both systolic BP and MAP were not significant (Additional file 1: Table S2). The size of the uranium effect estimate compared to other components may be due to its low ambient concentration (0.19 ng/m^3^), and relative IQR (0.2 ng/m^3^).

**Table 3:** Associations between major non-carbonaceous PM components and trace elements and diastolic blood pressure

**Component**	**Effect (mmHg)**	**SEM**	**p-value**	**Change per 1 ng/m3 CI (lower, upper)**	**IQR (ng)**
Uranium	15.22	7.44770	0.0434	2.29, 149.9	0.20
Rubium	0.146	0.05426	0.0081	0.0045, 0.0294	8.65
Crustal	0.076	0.03098	0.0153	5E-04, 0.0042	32.50
Manganese	0.006	0.00294	0.0465	3E-07, 4E-05	291.50
Aluminum	0.002	0.00098	0.0407	5E-08, 2E-06	1636.00
Potassium	0.0017	0.00072	0.0157	2E-07, 2E-06	1566.00
Magnesium	0.0014	0.00073	0.0487	5E-09, 2E-06	1590.00
Iron	0.0005	0.00023	0.0264	1E-08, 2E-07	4903.00

Data are expressed as change in response (effect) per IQR of pollutant, and per 1 ng/m^3^.

### Associations with carbon fractions

During all four exposure studies, PM_2.5_ EC concentration was consistently associated with increased HR with estimated changes of 11-32 bpm (4-11% increase) for a 1 μg/m^3^ increase in EC (Table 4). EC was also associated with 22-27% decreases in HRV during Studies 1 & 4. By comparison, OC and PM_2.5_ mass had little to no associations with cardiac automaticity.

**Table 4:** **Associations between PM**_**2.5 **_**mass, EC and OC and cardiac responses**

**Response**	**Study**	**Component**	**Change per IQR**	**SEM**	***p*-value**	**Change per 1 μg/m3 CI (lower, upper)**
**Heart rate**	1	PM_2.5_	2.757	1.502	0.068	--
		EC	7.059	0.885	<.0001	28.1, 36.1
		OC	-1.845	1.431	0.199	--
	2	PM_2.5_	1.343	1.635	0.412	--
		EC	8.354	1.454	<.0001	19.7, 28.1
		OC	0.879	1.107	0.428	--
	3	PM_2.5_	0.719	1.344	0.593	--
		EC	3.649	0.974	0.000	8.1, 14.1
		OC	-5.489	1.543	0.000	-2.4
	4	PM_2.5_	1.086	1.375	0.431	--
		EC	5.985	1.350	<.0001	20.1, 31.9
		OC	1.916	1.266	0.131	--
**lnSDNN**	1	PM_2.5_	0.094	0.026	0.000	0.0615, 0.0619
		EC	-0.031	0.018	0.086	--
		OC	0.001	0.026	0.955	--
	2	PM_2.5_	0.016	0.034	0.639	--
		EC	-0.061	0.031	0.053	--
		OC	-0.009	0.023	0.693	--
	3	PM_2.5_	0.024	0.029	0.415	--
		EC	0.006	0.020	0.774	--
		OC	-0.038	0.032	0.240	--
	4	PM_2.5_	0.022	0.027	0.406	--
		EC	-0.051	0.024	0.039	-0.2202, -0.2198
		OC	0.016	0.022	0.474	--
**lnRMSSD**	1	PM_2.5_	0.001	0.017	0.942	--
		EC	-0.048	0.011	<.0001	-0.267, -0.167
		OC	-0.011	0.016	0.494	--
	2	PM_2.5_	0.019	0.016	0.236	--
		EC	-0.023	0.015	0.127	--
		OC	0.007	0.011	0.541	--
	3	PM_2.5_	-0.009	0.016	0.603	--
		EC	-0.002	0.011	0.834	--
		OC	0.008	0.017	0.646	--
	4	PM_2.5_	-0.008	0.018	0.659	--
		EC	-0.039	0.017	0.022	-0.244, -0.096
		OC	0.020	0.015	0.193	--

Data are expressed as change in heart rate (bpm), lnSDNN (msec) and lnRMSSD (msec), per IQR of pollutant, and per change in 1 μg/m^3^ of pollutant.

Changes in blood pressure also appeared to be more influenced by variations in EC than by OC or PM mass (Table 5). Increases in systolic BP ranged from 3-14 mmHg (1.5-8% increases) for a 1 μg/m^3^ increase in EC for Studies 1-4. MAP was elevated by 4-9% in Studies 1 & 2 while diastolic BP increased significantly with EC by 4-10% in three of the four exposure studies.

**Table 5:** **Associations between PM**_**2.5**_**, EC and OC and vascular responses**

**Response**	**Study**	**Component**	**Change per IQR**	**SEM**	***p*-value**	**Change per 1 μg/m3 CI (lower, upper)**
**MAP**	1	PM_2.5_	0.576	0.897	0.521	--
		EC	2.894	0.536	<.0001	10.76, 15.64
		OC	-1.555	0.824	0.061	--
	2	PM_2.5_	3.183	0.827	0.000	0.775, 0.779
		EC	2.000	0.795	0.013	3.4, 7.8
		OC	1.099	0.573	0.056	--
	3	PM_2.5_	-0.661	0.604	0.276	--
		EC	0.369	0.411	0.370	--
		OC	-0.517	0.648	0.426	--
	4	PM_2.5_	-0.377	0.555	0.498	--
		EC	0.893	0.521	0.088	--
		OC	0.876	0.473	0.065	--
**Systolic**	1	PM_2.5_	0.454	0.992	0.647	--
		EC	3.219	0.594	<.0001	11.9, 17.3
		OC	-1.864	0.912	0.042	-2.5, -0.88
	2	PM_2.5_	3.392	0.903	0.000	0.825, 0.830
		EC	2.448	0.856	0.005	4.55, 9.45
		OC	1.208	0.620	0.053	--
	3	PM_2.5_	-0.268	0.673	0.690	--
		EC	0.986	0.453	0.031	1.6, 4.4
		OC	-1.462	0.715	0.042	-1.64, -0.55
	4	PM_2.5_	0.027	0.586	0.963	--
		EC	1.156	0.548	0.036	2.61, 7.38
		OC	0.532	0.502	0.290	--
**Diastolic**	1	PM_2.5_	0.767	0.826	0.354	--
		EC	2.720	0.492	<.0001	10.16, 14.64
		OC	-1.324	0.760	0.083	--
	2	PM_2.5_	3.071	0.788	0.000	0.747, 0.751
		EC	1.979	0.766	0.010	3.51, 7.89
		OC	1.181	0.552	0.033	0.727, 2.07
	3	PM_2.5_	-0.772	0.584	0.187	--
		EC	0.258	0.398	0.517	--
		OC	-0.097	0.628	0.878	--
	4	PM_2.5_	-0.404	0.569	0.479	--
		EC	1.195	0.523	0.023	2.93, 7.47
		OC	1.320	0.473	0.006	1.16, 2.44

Data are expressed as change in mean (MAP), systolic and diastolic pressures (mmHg) per IQR of pollutant, and per change in 1 μg/m^3^ of pollutant.

### Associations with carbon subfractions

**Figure 3:**
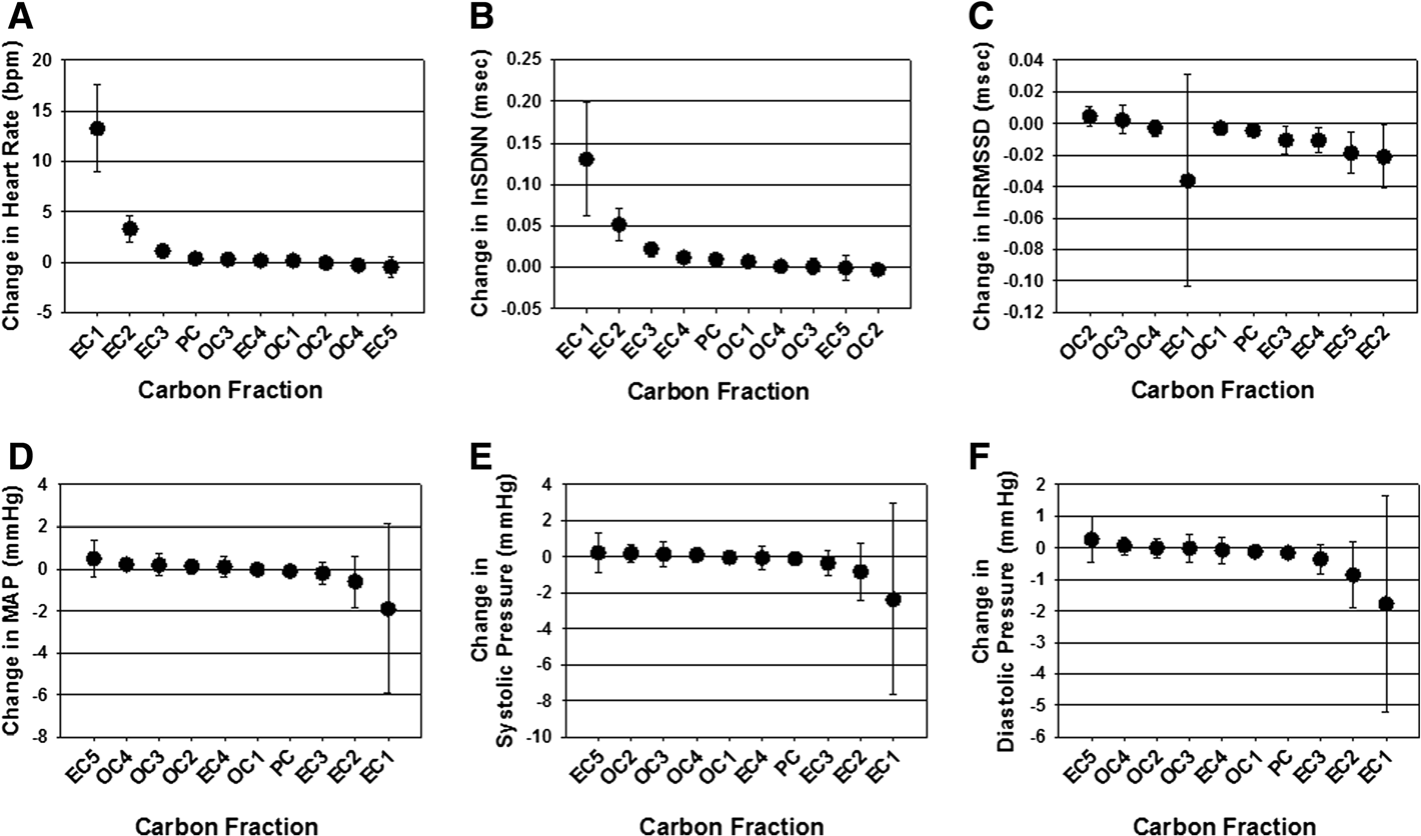
**Effect estimates for cardiovascular responses and PM**_**2.5 **_**carbon fractions and subfractions.** Data are expressed as change in heart rate **(A)**, HRV **(B,C)**, mean arterial BP **(D)**, systolic BP **(E)**, and diastolic BP **(F)** per IQR of carbon subfractions and were derived from combining all 8 h (daily) averages for each parameter from Studies 1-4. Estimates with confidence intervals that do not intersect the 0-axis are significant, *p* < 0.05.

Overall the daily concentrations of EC subfractions had greater associations with heart rate and HRV changes than OC subfractions (Figure 3A-C). Subfractions EC1, EC2, and EC3 were all significantly associated with increased heart rate and SDNN, with EC1 having the largest effect estimate (HR increase of 13 bpm; SDNN increase of approximately 7.5%). Interestingly, no effects were found for EC itself using the 8 h averages. EC2, EC3, EC4, and EC5 were all significantly negatively associated with reductions in RMSSD. EC1 showed extremely wide confidence intervals. Small effect estimates were seen for OC and OC1, the only organic carbon fractions with significant associations. Carbon subfractions had weak associations with vascular responses (Figure 3D-F). Except for total EC which had small effect estimates (>1 mmHg) for both MAP and DBP, no EC or OC subfractions were significantly associated with any change in blood pressure.

## Discussion

Results of this study clearly demonstrate that the EC and EC subfractions drive the acute changes in HR, HRV and BP in hypertensive rats exposed to an urban-industrial aerosol. These findings are consistent with our previous report of EC’s relation with HR in the same rat model [21], but we now extend these observations to identify EC-associated effects on BP as well, suggesting a more profound overall impact on cardiovascular health. Furthermore, we detected associations between EC subfractions and cardiac function, especially between EC1 and EC2 with HR and HRV. By comparison to EC, OC was associated with only modest and sometimes opposing cardiovascular effects in PM_2.5_-exposed rats. To our knowledge, our current findings are the first to describe associations of cardiovascular health effects with inhaled PM2.5 carbon subfractions using thermal/optical analytical methods.

Although not all source apportionment studies include EC and OC in their factor resolution, those that do typically find that these PM components load onto mobile source emissions factors [24,25]. Furthermore, the separation of carbonaceous fractions derived from progressive oxidation temperatures suggest that the subfraction EC1 is generally linked to diesel engine sources, whereas OC fractions (OC1-4) are normally associated with gasoline emissions [17,26]. It is notable, therefore, that the adverse cardiovascular responses we describe for EC and its subfractions are consistent with HR, HRV and BP responses reported during controlled exposures to whole diesel engine exhaust in the same SH strain of rats we used in the current study [27,28]. While major components of laboratory-generated diesel exhaust are gaseous inorganic compounds (nitrogen oxides, sulfur dioxide, and carbon monoxide), the minor components of particulate EC and of volatile and semivolatile organic compounds (OC) have been linked to stimulation of both sympathetic and parasympathetic cardiovascular effects in SH rats where exposures compared whole versus filtered exhaust [29,30]. A major difference in our field studies compared to laboratory-generated diesel engine exhaust is the atmospheric transformation of EC core particulates that might result in surface adherence of volatile organic hydrocarbons such as carbonyls, or in oxidative modifications that alter the particle’s toxicity [31,32]. OC was a major component of PM_2.5_ mass at our urban site, and though not strongly associated with health effects in our study, it may contribute to cardiovascular effects as a surface component of diesel soot particles.

Our study location in Dearborn, MI is located near automotive production industries with heavy vehicle and diesel traffic in the surrounding community. Of note are several active trucking facilities within a mile of the site, with several hundred trucks loading and unloading cargo daily, as well as a railyard within 250 m of the site. We recently compared cardiometabolic responses in twenty-five volunteers before, during and after ambient exposures at this same industrial site, relative to their residences in rural upwind areas of Dexter, MI [33]. After five daily exposures, decreases in HRV and insulin resistance were associated with increased PM2.5. Further analyses of source:health effect relationships found that changes in HR, BP and trends for impaired endothelial function were associated with the diesel source factor that impacted this site [34]. Fewer health effects were associated with other PM source factors, with motor vehicle sources being linked to changes in BP, and iron/steel and secondary aerosol source factors being associated with changes in HR. We recently reported dramatic drops in BP and HR in fructose-fed rats with cardiometabolic syndrome exposed to PM2.5 at this same site in Dearborn; however, analyses to attribute specific sources with CV responses have not been completed [35]. Our findings with EC and subfractions in the current study adds to our previous work with PM2.5-exposed SH rats where EC and traffic sources had robust associations with changes in HR and HRV in urban Detroit, MI and Steubenville, OH [20,21].

To date, carbon subfraction analysis has been employed in air pollution studies primarily to incorporate fractions into source apportionment analyses and, ideally, to improve source factor resolution [36,37]. For the most part, such studies have also been able to differentiate gasoline from diesel emissions, as discussed above. In addition, some work has investigated indoor and non-indoor sources of carbon fractions in residential homes [38]. We were able to identify one *in vitro* study including carbon subfractionation in which human lung epithelial cells were exposed to dust from soil and road surfaces in the western United States [39]. Release of inflammatory mediators was most highly correlated with the EC1 fraction, while lesser correlation coefficients were observed for OC fractions and pyrolized carbon. Compared to other EC fractions, compounds that comprise EC1 and EC2 would be more volatile, of smaller molecular weight, and a less complex structure. Less clear are the molecular targets, receptors or proteins with which different subfractions may preferentially interact to elicit biological responses. In addition to the greater responses induced in airway cells, smaller sized EC1 compounds would theoretically be better able to translocate and influence extarpulmonary responses. However, epidemiological or clinical evidence for EC subfraction-associated health effects is lacking, and we speculate that EC and EC1may be markers for other pollutants or pollutant mixtures that underlie the health effects we describe in exposed rats.

In similar PM_2.5_ field exposures in urban areas we have previously identified associations of health effects with a number of trace elements that are linked to industrial activities in the Midwest [21,40]. In the current analyses, several elements had statistically significant associations with HR, HRV and BP, but we interpret the effect sizes as having questionable biological relevance (e.g., < 0.1 bpm HR). Interestingly the greatest and most consistent effects were found with uranium, which had a considerable effect estimate for its association with increases in diastolic BP (13 mmHg). Using x-ray diffraction analysis to assess Detroit PM constituents, our colleagues showed that uranium is colocalized with EC in graphene structures [41]. Its source in the urban industrial airshed in southwest Michigan is unknown although it has been associated with coal combustion [42]. In our study, despite health effects associated with this element, other elements and components typically associated with coal-fired power plant emissions such as sulfate, selenium, and arsenic, yielded no adverse health effects findings.

Compared to most of our community-based animal studies, the current investigation is limited by the use of 8 h-integrated PM_2.5_ samples to estimate effects on daily changes in cardiovascular responses. Our group is unique in that we have used 30-minute sampling periods for both particle collection and cardiotelemetry recording; however, the necessary instrumentation was not available for all studies in this project. A second limitation is the lack of normotensive control subjects with which to compare our responses in the SH rats. We have previously used Wistar Kyoto rats as our healthy controls, but because of space limitations in the exposure chamber they were omitted to allow for a larger group number of hypertensive rats. As such any translation of our findings to understand potential susceptibility is limited.

In summary, this is the first report of cardiovascular health effects linked to inhalation exposure to ambient PM_2.5_ carbon subfractions. Increased BP and HR and decreases in HRV showed robust associations with EC, and our initial findings using thermal optical approaches yielded strong relationships of EC1 and EC2 with changes in HR and HRV. Interestingly, we found relatively fewer and weaker responses with OC fractions or trace elements. Black carbon has been proposed as an important indicator of PM-induced health effects [43], and our results with EC add to this evidence, specifically for adverse cardiovascular responses. Future research efforts that include the analyses of carbon subfractions are needed to confirm our observations, and will help to further characterize the contribution of EC to the health risk of PM_2.5_ exposures.

## Methods

### Animals

Sixty-four male spontaneously hypertensive (SH) rats 12-13 weeks of age (Charles River Laboratories, Portage, MI) were initially housed in animal facilities at Michigan State University (MSU) until moved to the mobile lab, where they were placed individually in polycarbonate cages on corn cob bedding with *ad libitum* access to food and water. Study protocols were approved by the Institutional Animal Care and Use Committee of MSU, an AAALAC accredited institution.

### Exposure to PM_2.5_

Inhalation exposures were conducted in AirCARE 1, a mobile air research laboratory parked at Salinas Elementary School in Dearborn, MI during the summers of 2009 and 2010. The site is located within 5 km of iron/steel production facilities, a coke oven, oil refinery, sewage sludge waste incinerator, a coal-fired power plant and major highways.

Concentrated PM_2.5_ was generated from ambient PM_2.5_ using a Harvard-type fine particle concentrator and whole body animal exposure chambers as previously described in detail [44]. Exposures were carried out in two stainless steel Hinners-type whole body inhalation chambers; one received PM2.5 while the other received HEPA-filtered clean air at the same flow rate as the experimental group. Eight SH rats were exposed in each chamber from 7:30 am – 3:30 pm for four consecutive days (Monday-Thursday). This exposure protocol was repeated on four separate occasions, with four separate cohorts of animals in different weeks: Study 1 (August 10-13, 2009), Study 2 (August 17-20, 2009) Study 3 (July 12 – 15, 2010) and Study 4 (July 19 -22, 2010). After each 8 h exposure, animals were removed from chambers and returned to their cages.

### Exposure characterization

Chamber PM_2.5_ samples were collected on Teflon and Quartz filters (Gelman Sciences, Ann Arbor MI) by attaching Teflon filter packs to the back of the animal exposure chamber with a flow rate of 3 LPM for the duration of each 8-h exposure period. Both ambient and concentrated PM_2.5_ mass concentrations were measured continuously using a Tapered Element Oscillating Microbalance (TEOM). Annular denuder/filter packs were employed to collect major inorganic fine particulate ions. Gravimetric determinations were made using a microbalance (MT-5 Mettler Toledo, Columbus OH) in a temperature/humidity-controlled Class 100 clean laboratory and followed Federal Reference Method (USEPA 1997). PM samples collected on quartz filters were analyzed for carbonaceous aerosols by a thermal-optical analyzer using the NIOSH method (Sunset Labs, Forest Grove, OR). Annular denuder/filter pack samples were analyzed for major ions by ion chromatography (Model ICS-90, DIONEX, Sunnyvale, CA). PM samples collected on Teflon filters were analyzed for a suite of trace elements using inductively coupled plasma-mass spectrometry (ICP-MS) (ELEMENT2, Thermo Finnigan, San Jose, CA).

### Cardiovascular telemetry

Two weeks before exposures, animals were surgically implanted with PhysioTel Multiplus transmitters (# C50-PXT; Data Sciences International; DSI, St. Paul, MN) that emit radio signals of electrocardiograms (ECG) and blood pressure (BP). Transmitters were placed with ECG leads terminating in a Lead II configuration to sample cardiac parameters and the pressure catheter placed in the aorta via the femoral artery. Telemetry receivers (RLA3000, DSI) were modified and affixed inside individual cages in exposure chambers that were customized for telemetry studies. Datastreams of 30 second duration were collected and analyzed every 5 minutes during exposures. Automated ECG analysis (DSI, ART3.2) allowed for R-wave detection on a beat-to-beat basis. The R-R intervals for all normal beats (N-N intervals) were used to calculate HR and time-domain measures of HRV: standard deviation of the normal-to-normal intervals (SDNN), an indicator of overall autonomic tone, and the square root of the mean squared differences of successive normal-to-normal intervals (RMSSD), an estimate of parasympathetic tone.

### Statistical analyses

To determine exposure –related differences in cardiac and vascular indices we used mixed model analyses using SAS (Version 9.2, Cary, NC); this approach accounts for the longitudinal nature of the measurements on each animal. To reduce the skewness of the HRV measures, we natural log–transformed the SDNN and RMSSD after adding 1. Analyses comparing Air- vs PM_2.5_ -exposed rats (Table 2), and the associations between PM_2.5_, EC and OC and cardiovascular responses (Tables 4 and 5) were derived from 1-hour samples of both PM and health effect responses during each of the four field exposure studies. Data for trace elements and carbon subfractions and their associations with health responses (Table 3, Figure 3, Additional file 1: Tables S1 and S2) were derived from 8-hour samples and integrated across the four field exposures (*n* = 32 samples). The criterion for significance was set at *p* ≤ 0.05 for all parameters.

## Abbreviations

BP: Blood pressure; EC: Elemental carbon; HR: Heart rate; MAP: Mean arterial pressure; OC: Organic carbon; PM_2.5_: Fine particulate matter; RMSSD: Root mean square of successive differences of adjacent interbeat intervals; SDNN: Standard deviation between normal-to-normal heart beats.

## Competing interests

The authors declare they have no actual or potential competing interests.

## Authors’ contributions

JGW developed the study design, carried out the animal experiments and the collection, analysis and interpretation of data, and drafted the manuscript. ASK conducted data analysis and interpretation. MM conducted the collection and characterization of PM2.5 and components, and helped draft the manuscript. JTD oversaw the field site operations, community engagement and helped draft the manuscript. JRH developed the study design and directed field exposures. ACR assisted with study design, data interpretation and helped draft the manuscript. All authors read and approved the final manuscript.

## Supplementary Material

Additional file 1: Table S1 Effect of Major Component and Trace Elements on Cardiac Responses. Data are expressed as change in response per IQR of pollutant. PM2.5 components with significant effects are indicated in bold. **Table S2.** Effect of Major Component and Trace Elements on Vascular Responses. Data are expressed as change in response per IQR of pollutant. PM2.5 components with significant effects are indicated in bold. Click here for file
