# Supplementary material for: PM2.5-induced cardiovascular dysregulation in rats is associated with elemental carbon and temperature-resolved carbon subfractions
Source: Part Fibre Toxicol. 2014 May 22;11:25. doi: 10.1186/1743-8977-11-25 (PMC4051889; doi:10.1186/1743-8977-11-25)
Supplement: Additional file 1: Table S1 — Effect of Major Component and Trace Elements on Cardiac Responses. Data are expressed as change in response per IQR of pollutant. PM2.5 components with significant effects are indicated in bold. Table S2. Effect of Major Component and Trace Elements on Vascular Responses. Data are expressed as change in response per IQR of pollutant. PM2.5 components with significant effects are indicated in bold. [file 1743-8977-11-25-S1.docx]

**Table S1. Effect of Major Component and Trace Elements on Cardiac Responses.** Data are expressed as change in response per IQR of pollutant. PM2.5 components with significant effects are indicated in bold.

**Table S2. Effect of Major Component and Trace Elements on Vascular Responses.** Data are expressed as change in response per IQR of pollutant. PM2.5 components with significant effects are indicated in bold.
